# Lorcaserin and phentermine exert anti-obesity effects with modulation of the gut microbiota

**DOI:** 10.3389/fmicb.2022.1109651

**Published:** 2023-01-05

**Authors:** Eun-Ji Song, Na Rae Shin, Songhee Jeon, Young-Do Nam, Hojun Kim

**Affiliations:** ^1^Research Group of Personalized Diet, Korea Food Research Institute, Iseo-myeon, South Korea; ^2^Department of Rehabilitation Medicine of Korean Medicine, Dongguk University, Goyang-si, South Korea; ^3^Department of Biomedical Sciences, Center for Global Future Biomedical Scientists at Chonnam National University, Gwangju, South Korea

**Keywords:** obesity, anti-obesity drug, gut microbiota, lorcaserin, phentermine

## Abstract

Although drugs have been reported to modulate the gut microbiota, the effects of anti-obesity drugs on the gut microbiota remain unclear. Lorcaserin (LS) and phentermine (PT) are commonly used anti-obesity drugs. However, to our best knowledge, no studies have simultaneously assessed the effects of LS and PT on obesity and gut microbiota. This study aimed to explore the relationship between the anti-obesity effects of LS and PT and re-modulation of host gut microbiota. To test hypothesis, we fed C57BL/6J mice with a high-fat diet supplemented with LS and PT *via* oral gavage for 8 weeks. After sacrifice, body weight, fat accumulation, and serum biomarkers were measured, and the gut microbial composition was analyzed using 16 s rRNA amplicon sequencing. LS and PT were observed to modulate the gut microbial composition and restore gut microbial dysbiosis, as indicated by an increased *Firmicutes/Bacteroidetes* ratio. Significantly modulated genera by LS and PT treatment were strongly correlated with obesity-related markers. Additionally, LS and PT increased the mRNA level of G protein-coupled receptor 120 (GPR120) in the colon tissue. ASV3566, which corresponds to *Eubacterium coprostanoligenes*, was correlated with GPR120 and obesity-related markers such as glutamic pyruvic transaminase (GPT) and serum triglyceride (TG). In conclusion, LS and PT can modulate the gut microbiota dysbiosis and the gut microbiota plays a role in mediating the anti-obesity effect of drugs.

## 1. Introduction

Obesity has become a global public health problem induced by excessive body fat due to increased energy intake and reduced expenditure. Obesity increases the risk of chronic metabolic diseases, such as type 2 diabetes (T2D), cardiovascular disease, and chronic low-grade inflammation; therefore, effective management strategies are needed (Vijay-Kumar et al., 2010). Recent studies have shown that obesity and obesity-induced metabolic disorders are closely related to the gut microbiota (Zhao, 2013; Muscogiuri et al., 2019). The composition of the gut microbiota and obesity influence each other, and indicators of dysbiosis such as the *Firmicutes*/*Bacteroidetes* (F/B) ratio are also markers of obesity (Ley et al., 2006; Boulangé et al., 2016). Moreover, microbial metabolites such as short-chain fatty acids (SCFAs) also affect obesity and host metabolism, including glycemic control, fat metabolism, and regulation of inflammation and immunity (van der Hee and Wells, 2021).

Recently, the role of the gut microbiota in drug treatment for obesity has been considered since the gut microbiota is involved in drug response and metabolism (Wilson and Nicholson, 2009; Kang et al., 2013; Vila et al., 2020). Moreover, several studies have reported alterations in the gut microbiota following drug treatment in clinical trials (Pascale et al., 2019; Vila et al., 2020). Metformin, a first-line clinical medicine for T2DM, enhanced the relative abundance of *Akkermansia*, which has the potential for glucose metabolism and insulin modulation (Shin et al., 2014; Montandon and Jornayvaz, 2017). Nuciferine has the ability to reduce high-fat diet (HFD)-induced obesity by altering the relative abundance of the gut microbiota, which is related to SCFA production and metabolic parameters (Wang et al., 2020). Orlistat modified the gut microbial composition, and the increased bacterial species was correlated with several metabolic pathways (Ke et al., 2020). Lorcaserin (LS) and phentermine (PT) are commonly used anti-obesity drugs. LS is a serotonin agent that selectively stimulates serotonin 2C receptor agonist, and PT modulates catecholamines in the hypothalamus, reducing appetite (Kim et al., 2014). However, to our knowledge, no studies have been published on the effects of LS and PT on gut microbiota. In this study, we aimed to investigate the impact of LS and PT on the composition of the gut microbiota in a HFD model. This research may provide support for the application of LS and PT in the treatment of obesity.

## 2. Materials and methods

### 2.1. Animal studies

Six-week-old male C57BL/6 mice obtained from DBL Inc. (Eumseong-gun, South Korea) were acclimatized for 1 week under a controlled environment with a 12-h light/dark cycle at 25°C and 50–60% relative humidity with a chow diet (Purina Irradiated Laboratory Rodent Chow, Purina Korea, Seoul, South Korea). The animal study was approved by the Institutional Animal Care and Use Committee (IACUC-2019-06187) of Dongguk University Ilsan Hospital and performed according to the Guide for the Care and Use of Laboratory Animals (National Research Council, 2011).

To assess the efficacy of anti-obesity drug treatment in HFD-induced obese mice, mice were randomly divided into four groups: control group (NOR), HFD, HFD + LS, and HFD + PT; *n* = 7–8 per group. The mice in NOR were fed a 10% fat control diet (D12450B, Research Diets Inc., United States), and other mice were fed a 60% HFD (D12492, Research Diets Inc., New Brunswick, NJ, United States) for 4 weeks. Moreover, the mice in the HFD + LS and HFD + PT groups received LS (10 mg/kg/day) and PT (5 mg/kg/day), respectively, *via* oral gavage. The mice in the NOR and HFD groups, which were untreated, were orally administered with water. The treatments were administered five times a week for 8 weeks.

Body weight was measured once a week. Food intake was measured three times a week per cage, and was expressed as the average daily food intake per mouse. After the termination of the experimental period, all animals were subjected to overnight fasting. The animals were then sacrificed with a combination of Zoletil (tiletamine-zolazepam, Virbac, Carros, France) and Rompun (xylazine-hydrochloride, Bayer, Leverkusen, Germany; 1:1, *v*/*v*). Subsequently, blood samples were collected from the central aorta and rapidly transferred into a BD Vacutainer (BD, Franklin Lakes, NJ, United States). The blood samples were allowed to clot for 2 h at room temperature and then centrifuged at 3000 × *g* for 15 min. The sera were separated and stored at −80°C for further analyses. The visceral adipose tissue, perigonadal adipose tissues, and colon tissues were excised quickly, washed in ice-cold PBS (pH 7.4), dried, weighed, and stored at −80°C until use. The data for the NOR and HFD groups were retrieved from a simultaneous study by Song et al. (2022).

### 2.2. Serum biochemical analyses and oral glucose tolerance test (OGTT)

Serum triglyceride (TG), total cholesterol (TC), and glutamic pyruvic transaminase (GPT) levels were determined using commercial enzymatic assay kits (Asan Pharmaceutical Co., Seoul, South Korea). Serum insulin concentration was measured using an Ultra-Sensitive Mouse Insulin ELISA kit (MIOBS, Yokohama, Japan) according to the manufacturer’s instructions. For the OGTT, the animals were subjected to overnight (12 h) fasting and administered sterilized glucose solution (2 g/kg, Sigma, Louis, MO, United States) *via* oral gavage. The glucose levels in the blood samples collected from the tail vein were measured at five different time points (0, 30, 60, 90, and 120 min) using the ACCU-CHEK Active glucometer (ACCU-CHEK, Mannheim, Germany).

### 2.3. Quantitative real-time PCR analysis

Total RNA was extracted from colonic tissue using TRIzol (Invitrogen Life Technologies, Carlsbad, CA, United States) according to the manufacturer’s instructions. The quality and quantity of crude RNA were examined using a NanoPhotometer (P330-IMPLEN, Munich, Germany). RNA was stored at −80°C for further analysis using RT-qPCR. An equal quantity of each RNA sample was reverse transcribed using an oligo deoxythymine 18-mer primer (Thermo Scientific, Waltham, MA, United States), and cDNA was synthesized using a cDNA RT PreMix kit (Bioneer, Daejeon, South Korea). Quantitative analysis of the mRNA expression of GPR41, GPR43, GPR120, IL-10, IL-13, TNF-α, IL-1β, IL-6, MCP-1 and glyceraldehyde-3-phosphatase dehydrogenase (GAPDH) was performed using RT-qPCR with a Light Cycler 480^™^ platform (Roche, Roche Applied Science, Mannheim, Germany) using a SYBR Green Real-time PCR Master Mix (Toyobo, Osaka, Japan). Data processing and analysis were performed using a dedicated Light Cycler software (Roche Applied Science, version 1.2). The Ct values were normalized using GAPDH, and the relative gene expression was quantified using the standard 2^−△△Ct^ method. Primer sequences are listed in Supplementary Table 1.

### 2.4. Sequence-based gut microbiota analysis

Fresh fecal samples were collected 1 week before sacrifice and stored at −80°C until use. Genomic DNA was isolated from the samples using the QIAamp stool DNA mini kit (QIAGEN, Hilden, Germany) according to the manufacturer’s instructions. PCR amplification of the V1–V2 region of the 16S rRNA gene was performed using a thermal cycler system (Bio-Rad, Hercules, CA, United States). Then, sequencing reactions were performed using the Ion Torrent Personal Genome Machine (Ion PGM, Thermo Scientific, Wilmington, DE, United States) according to the manufacturer’s instructions. Raw sequence reads were quality-filtered, and quality-controlled reads were processed for diversity analysis and taxonomy assignment using the Quantitative Insights into the Microbial Ecology 2 (QIIME2) software package (Caporaso et al., 2010). All raw sequencing data described in this study are available at the DNA Data Bank of Japan (accession number DRA013284).

### 2.5. Statistical analysis

Significance was evaluated using one-way analysis of variance (ANOVA) followed by Dunnett’s multiple comparisons or Kruskal–Wallis test followed by Dunn’s test. Significance was set at *p* ≤ 0.05. To identify taxa with differing relative abundances among groups, the linear discriminant analysis effect size (LEfSe) method was conducted using a web-based program (Segata et al., 2011).^1^ The strength of the relationships between parameters was assessed using Spearman’s correlation test.

## 3. Results

### 3.1. Effects of anti-obesity drugs on HFD-induced obesity

**Figure 1:**
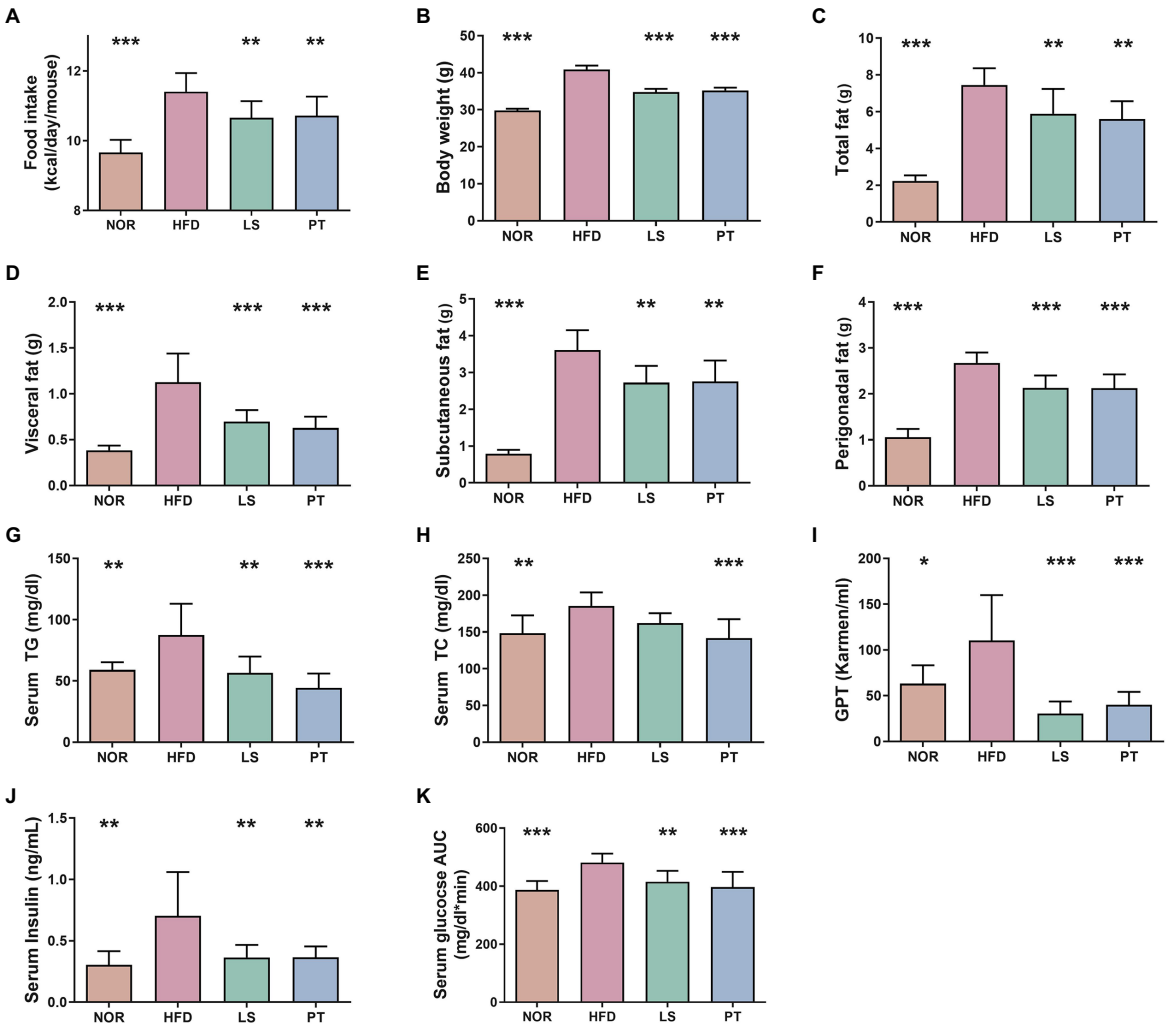
Effect of anti-obesity drugs on weight, serum biomarker, and glucose tolerance in a high-fat diet (HFD)-induced obese model after treatment for 8 weeks. **(A)** Food intake **(B)** Body weight. **(C)** Total fat. **(D)** Visceral fat. **(E)** Subcutaneous fat. **(F)** Perigonadal fat. **(G)** Serum triglyceride (TG). **(H)** Serum total cholesterol (TC). **(I)** Serum glutamic pyruvic transaminase (GPT). **(J)** Serum insulin. **(K)** Oral glucose tolerance test (OGTT) AUC. Data are expressed as the mean ± SEM. Statistical significance was assessed using one-way ANOVA. ^*^p < 0.05, ^**^p < 0.01, ^***^p < 0.001, vs. HFD. NOR, control group; LS, lorcaserin; PT, phentermine.

To investigate the effects of anti-obesity drugs, we established an obese mouse model by feeding them HFD for 4 weeks, followed by treatment with LS or PT for 8 weeks. HFD led to significant increases in food intake, body weight, and fat accumulation compared to the NOR group (Figures 1A–F).

Lorcaserin and PT supplementation significantly decreased food intake compared with HFD (Figure 1A). Notably, LS and PT supplementation affected body weight and fat accumulation upon HFD feeding. LS and PT supplementation significantly decreased body weight compared with that in the HFD group (Figure 1B). In addition, LS and PT supplementation significantly decreased weights of total fat, visceral fat, subcutaneous fat, and perigonadal fat (Figures 1C–F).

The anti-obesity drugs also influenced serum biochemical parameters, such as TG, TC, GPT, insulin, OGTT levels, and fasting glucose (Figures 1G–K). HFD led to significant increases in all serum biochemical parameters compared to the NOR group. Compared to the HFD group, the LS group had significantly reduced serum biochemical parameters, except for TC. Furthermore, the PT group had significantly reduced all serum biochemical parameters compared to the HFD group.

### 3.2. Anti-obesity drugs altered the composition of the gut microbiota

To assess the effect of anti-obesity drugs on the gut microbiota, 16S rRNA amplicon sequencing was performed using fecal samples. A total of 6,370,426 reads were generated, and a total of 3,417,057 high-quality reads were obtained through quality control, such as denoising and chimera removal. For subsequent analysis, 25,795 reads per sample were used through even subsampling.

**Figure 2:**
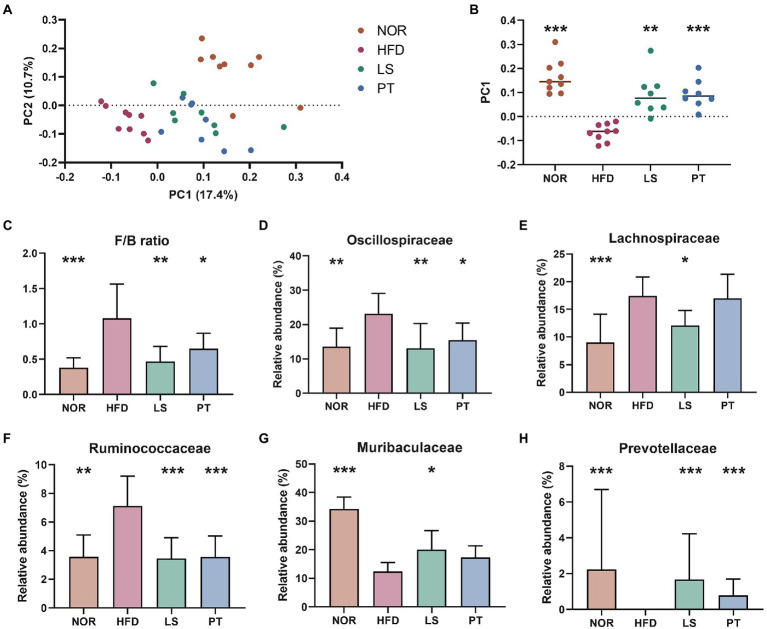
Effect of anti-obesity drugs on the structure of the gut microbiota in a high-fat diet (HFD)-induced obese model after treatment for 8 weeks. **(A)** Unweighted UniFrac PCoA plot based on the ASV abundance of each mouse. **(B)** Box plot of PC1 values in each group. **(C)** The F/B ratio was calculated as a biomarker of gut dysbiosis. **(D–H)** The relative abundance at the family level significantly changes with HFD. Values are presented as the mean ± SEM. Statistical significance was assessed using the Kruskal–Wallis test. ^*^p < 0.05, ^**^p < 0.01, ^***^p < 0.001, vs. HFD. NOR, control group; LS, lorcaserin; PT, phentermine.

Beta diversity analysis was performed to evaluate the overall structural changes in the gut microbiota. The resulting of PCoA plot showed separation among groups, and significance was determined using the PC1 value on the unweighted UniFrac distance matrix (Figures 2A, B). LS and PT treatment resulted in a significant structural shift along with the PC1 values from the HFD group to the direction of the NOR group. The resulting of PLS-DA plot at phylum level also showed separation among groups, and the taxa with greater absolute values on component 1 was visualized using a bar plot (Supplementary Figure 1). The most relevant original variables were *Firmicutes* and *Bacteroides*. LS and PT treatments significantly restored the F/B ratio (Figure 2C).

At family level, the resulting of PLS-DA plot showed clear separation among groups (Supplementary Figures 2A–D). Of note, PT treatment showed a return to NOR group. The taxa with greater absolute values on component 1 was visualized using a bar plot (Supplementary Figures 2E–G). The most relevant original variables were *Lachnospiraceae*, *Muribaculaceae*, *Oscillospiraceae*, *Prevotellaceae*, *Ruminococcaceae*, and *Streptococcaceae*. Statistical test revealed a significant increase in *Oscillospiraceae*, *Lachnospiraceae*, and *Ruminococcaceae*, and a decrease in the abundance of *Muribaculaceae* and *Prevotellaceae* in the HFD group compared to those in the NOR group (Figures 2D–H). LS treatment significantly restored these taxa to levels similar to those in the NOR group. PT treatment significantly restored the levels of *Oscillospiraceae*, *Ruminococcaceae*, and *Prevotellaceae*.

**Figure 3:**
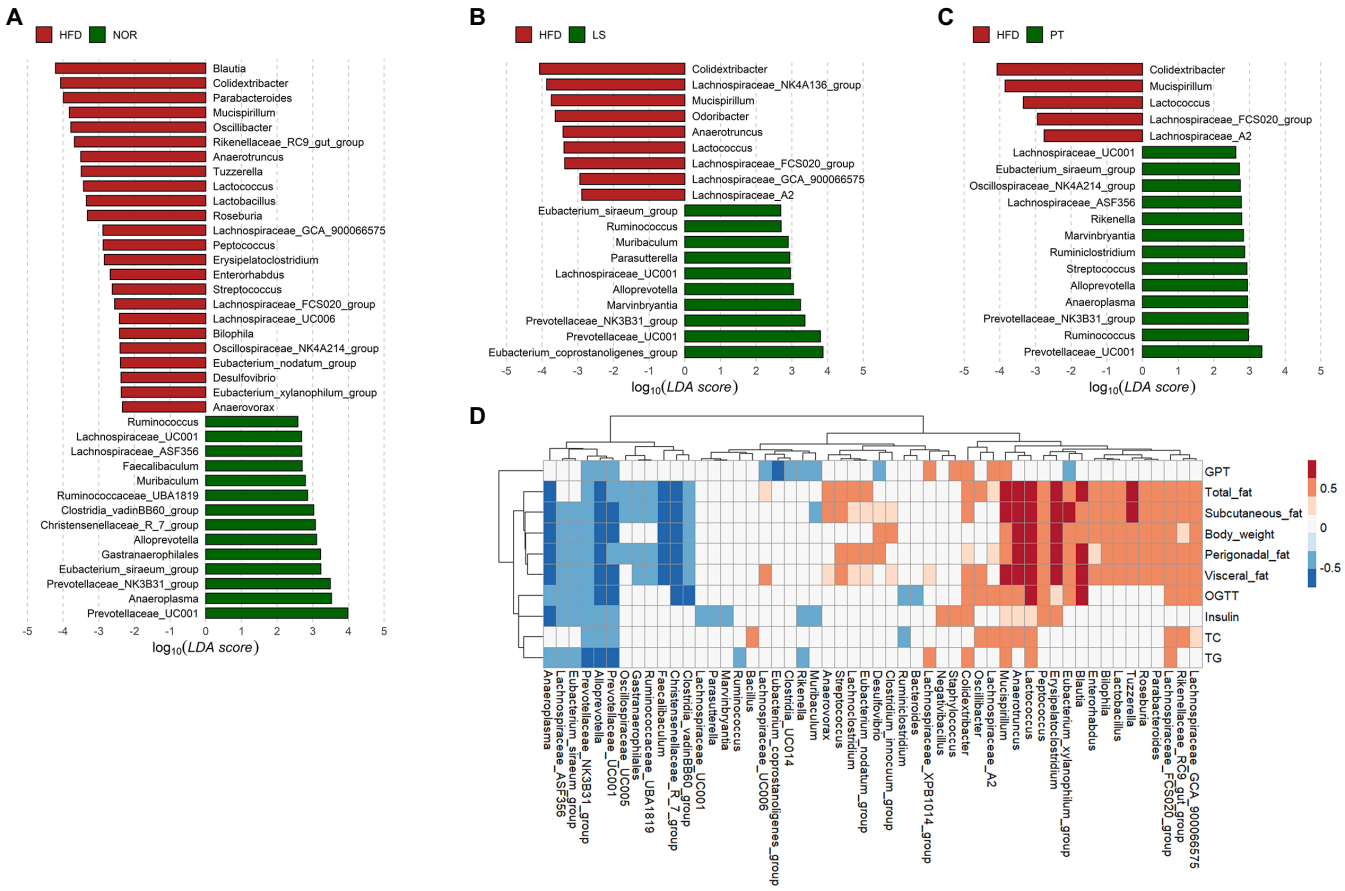
Association of microbiota with anti-obesity drugs and obesity-related parameters. **(A–C)** LEfSe analysis identifies the significant differently abundant taxa with a cutoff value of log_10_(LDA score) above 2.0. **(A)** control group (NOR)-, **(B)** lorcaserin (LS)-, and **(C)** phentermine (PT)-enriched taxa are indicated with a positive LDA score (green), while taxa enriched in high-fat diet (HFD) are characterized by a negative score (red). **(D)** A heatmap generated using Spearman correlation shows the association of significantly differential taxa among groups from the LEfSe results with obesity-related markers. Only correlations with a significance *p* < 0.05 are represented. Color scale indicates the value of the correlation coefficient.

At genus level, the resulting of PLS-DA plot at family level showed clear separation among groups (Supplementary Figures 3A–D). Of note, PT treatment showed a return to NOR group. The taxa with greater absolute values on component 1 was visualized using a bar plot (Supplementary Figures 3E–G). LEfSe was performed to identify significantly different taxa between the HFD groups and NOR or drug treatment groups (Figure 3). A total of 39 genera were significantly different between the NOR and the HFD groups. Compared to the HFD group, 13 and 12 genera in the LS and PT groups, respectively, matched those in the NOR group. Notably, LS and PT treatment restored the abundance of *Prevotellaceae_UC001*, *Prevotellaceae_NK3B31_group,* and *Alloprevotella* and increased the level of *Colidextribacter* and *Mucispirillum* due to HFD compared to the NOR group (Figures 3A–C).

Spearman’s rank correlations were used to identify the genera that were strongly correlated with obesity-related markers. Most of the significantly different taxa from the LEfSe results showed a significant correlation with obesity-related markers (Figure 3D). Notably, *Prevotellaceae_UC001*, *Prevotellaceae_NK3B31_group*, and *Alloprevotella* were significantly negatively correlated with all obesity-related markers. In addition, *Mucispirillum* exhibited a significant positive correlation with all obesity-related markers. *Colidextribacter* showed a significant positive correlation with fat accumulation, insulin, TG, and GPT.

### 3.3. Effects of anti-obesity drugs on GPRs and its association with the gut microbiota

**Figure 4:**
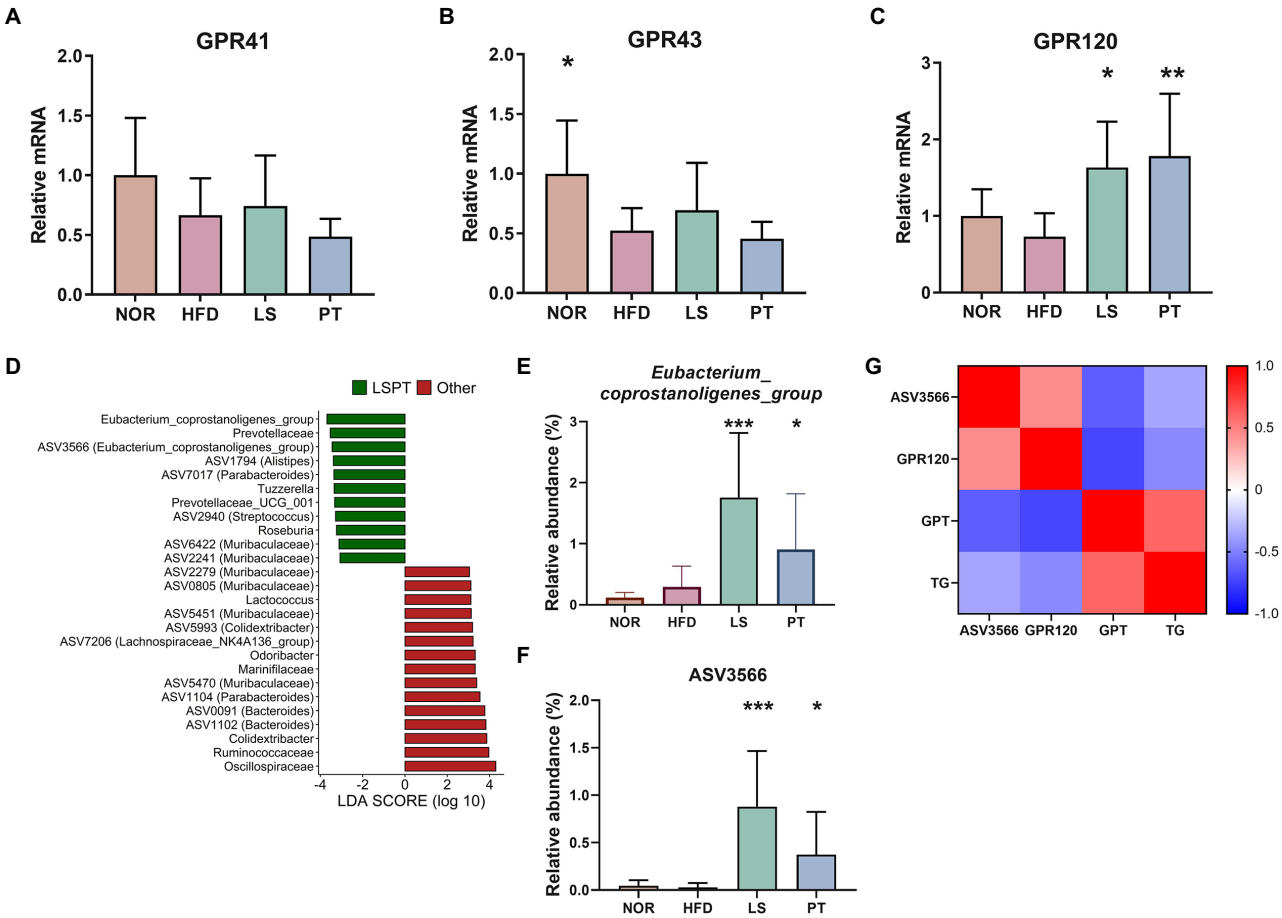
Effect of anti-obesity drugs on GPRs in colonic tissue and the association of the gut microbiota and GPRs. **(A–C)** The mRNA gene expression of GPRs in high-fat diet (HFD)-induced obese model. **(D)** LEfSe plot of gut microbiota at the genus and ASV levels in the comparison between LSPT (LS + PT) and others (NOR + HFD). The relative abundance of **(E)** *Eubacterium_coprostanoligenes_group* and **(F)** ASV3566 in each group. **(G)** A heatmap generated using Spearman correlation shows associations of ASV3566 with GPR120, GPT, and TG. The color scale indicates the value of the correlation coefficient. Data are expressed as the mean ± SEM. Statistical significance was assessed using one-way ANOVA. ^*^p < 0.05, ^**^p < 0.01, ^***^p < 0.001, vs. HFD. HFD, high-fat diet; NOR, control group; LS, lorcaserin; PT, phentermine.

G protein-coupled receptors (GPRs) are mediators of host-microbial interactions (Cohen et al., 2017; Melhem et al., 2019). To determine whether GPRs are involved in the effects of anti-obesity drugs and the effect of gut microbiota, we investigated the effect of anti-obesity drugs on the gene expressions of GPRs in colonic tissues (Figure 4). Anti-obesity drugs did not affect the gene expression of *GPR41* and *GPR43* (Figures 4A, B). Although no significant difference was observed between the NOR group and the HFD group, *GPR120* was significantly increased after treatment with LS and PT, as compared with the HFD group (Figure 4C).

Furthermore, we identified the microorganisms correlated with GPR120, which was a result of the LS and PT treatment. First, we performed the LEfSe analysis to detect microorganisms increased by LS and PT. Results comparing LS and PT groups with other groups showed that *Eubacterium_coprostanoligenes_group* had the highest LDA score (Figure 4D). The relative abundance of *Eubacterium_coprostanoligenes_group* was significantly higher in the LS and PT groups than in the NOR and HFD groups (Figure 4E). ASV3566, which corresponds to *Eubacterium coprostanoligenes*, was rare in other groups but was significantly abundant in LS and PT groups (Figure 4F). Next, using Spearman’s correlation analysis, we found that ASV3566 had a significant positive correlation with GPR120 and a significant negative correlation with GPT and TG (Figure 4G). Moreover, GPR120 had a significant negative correlation with GPR and TG.

GPR120 is known to regulate adipogenesis, inflammation, glucose uptake, and insulin resistance (Song et al., 2017). Therefore, we next investigated the regulation of adipose inflammation by LS and PT (Supplementary Figure 4). IL-10, which is anti-inflammatory cytokine, was not significantly increased by LS and PT treatment in the HFD model (Supplementary Figure 4A). However, IL-13, which is anti-inflammatory cytokine, was significantly increased by LS and PT treatment in the HFD model (Supplementary Figure 4B). Moreover, the pro-inflammatory cytokines TNF-a and IL-1b were significantly decreased by LS treatment in the HFD model (Supplementary Figures 4C, D). Notably, the pro-inflammatory cytokines IL-6 and MCP-1 were significantly decreased by LS and PT treatment in the HFD model (Supplementary Figures 4E, F).

## 4. Discussion

In this study, we hypothesized that anti-obesity drugs such as LS and PT induce changes in the gut microbiota. Our study revealed that drug supplementation can prevent HFD-induced obesity and restore gut microbial dysbiosis. Modulated members of the gut microbiota correlated with obesity-related markers. Furthermore, drug supplementation elevated the mRNA level of *GPR120* and relieved adipose inflammation. Notably, we found the association of *Eubacterium coprostanoligenes* ASV3566 with GPR120 and TG. These results indicate that LS and PT can modulate the gut microbiota dysbiosis and the gut microbiota plays a role in mediating the anti-obesity effect of drugs.

A previous study reported that two types of gut microbes, *Firmicutes* and *Bacteroidetes*, were predominant in the human gut, and a high population of *Bacteroidetes* was observed in obese people (Ley et al., 2006). Since then, the F/B ratio has been used as a marker of gut microbial dysbiosis and as an indicator in obesity research (Indiani et al., 2018; Stojanov et al., 2020; Palmas et al., 2021). In our study, LS and PT significantly reduced the increased F/B ratio due to HFD feeding. Other studies have shown the results of drug-induced anti-obesity effects and reduction in the F/B ratio. Wang et al. (2020) showed that nuciferine supplementation significantly lowered the F/B ratio increased by HFD in an obese rat model. Ke et al. (2020) showed that orlistat treatment decreased the F/B ratio in an obese mouse model. Furthermore, An et al. (2018) showed that treatment with cordycepin, a major bioactive component from *Cordyceps militaris*, prevented body weight gain and decreased the F/B ratio in HFD-induced obese rats. These findings were consistent with our results. The increase in *Firmicutes* is linked to an increase in the breakdown of indigestible polysaccharides and the production of SCFAs. SCFAs are the primary end-products of bacterial fermentation of non-digestible carbohydrates in the gut that have an impact on host metabolism, such as gut integrity, glucose homeostasis, lipid metabolism, appetite regulation, and immune function (Morrison and Preston, 2016). However, SCFAs act as an energy source, and an excessive supply of SCFAs can lead to an increase in energy harvest (Murphy et al., 2010). Several studies have shown an increase in energy harvesting along with an increase in fecal SCFAs in obese individuals (Turnbaugh et al., 2006; Schwiertz et al., 2010; Fernandes et al., 2014; Rahat-Rozenbloom et al., 2014; Goffredo et al., 2016).

Furthermore, we found that the relative abundance of *Oscillospiraceae*, *Lachnospiraceae*, and *Ruminococcaceae* in the *Firmicutes* phylum was significantly increased by HFD. An increased *Oscillospiraceae* abundance is not commonly observed after an HFD or in obese individuals. However, some reports have shown that obesity increases *Lachnospiraceae* and *Ruminococcaceae*. Zeng et al. (2016) demonstrated that an increase in the abundance of *Lachnospiraceae* was accompanied by inflammation in HFD-fed mice. Colonization of *Lachnospiraceae* in germ-free *ob/ob* mice induced significant increases in mesenteric adipose tissue weight (Kameyama and Itoh, 2014). A study on Mexican children suggested that the abundance of *Lachnospiraceae* was significantly enhanced in overweight and obese individuals (Murugesan et al., 2015). Another study on Mexican women observed a significant increase in the abundance of *Lachnospiraceae* and *Ruminococcaceae* in individuals with obesity alone and obesity with metabolic syndrome (Chávez-Carbajal et al., 2019). An Italian cohort study showed that *Lachnospiraceae* and *Ruminococcus gnavus* (belonging to the *Ruminococcaceae* family) were significantly increased in overweight and obese patients and were positively correlated with body fat (Palmas et al., 2021). These results are consistent with our results. Moreover, LS and PT supplementation significantly restored the HFD-induced increase in *Lachnospiraceae* and *Ruminococcaceae*. We also observed that the relative abundance of *Prevotellaceae* and its genera, such as *Prevotellaceae_UCG_001*, *Prevotellaceae_NK3B31_group*, and *Alloprevotella*, was significantly decreased by HFD. Moreover, we observed that the altered levels of these taxa were recovered by LS and PT supplementation. *Prevotellaceae_UCG_001* was significantly decreased in *ob/ob* mice and enriched by prebiotic inulin treatment. In addition, *Prevotellaceae_UCG_001* had a significant negative correlation with liver indices and TC/HFDL-C and a significant positive correlation with the AMPK signaling pathways, which have downstream metabolic functions that promote fatty acid metabolism and glycolysis and inhibit hepatic fatty acid synthesis, cholesterol synthesis, and gluconeogenesis (Song et al., 2019). *Prevotellaceae_NK3B31_group* and *Alloprevotella* were notably decreased in the HFD group, and these changes were reversed by polyphenolic-enriched peach peel extract in the HFD mouse model (Kan et al., 2020). Concurringly, LS and PT supplementation significantly restored the decreased levels of *Prevotellaceae* and *Alloprevotella* by HFD. Moreover, correlation analysis showed that *Prevotellaceae_UC001*, *Prevotellaceae_NK3B31_group*, and *Alloprevotella* were significantly negatively correlated with obesity-related markers, including body weight, fat accumulation, and metabolic parameters. These results suggest that the recovery of HFD-induced microbial dysbiosis is linked to the prevention of obesity by drugs. Therefore, we postulate that changes in the gut microbiota caused by LS and PT supplementation may have a positive anti-obesity effect.

Additionally, LS and PT recovered the HFD-induced increase in the level of *Colidextribacter*. *Colidextribacter* was positively correlated with fat accumulation, insulin, TG, and GPT. *Colidextribacter* was identified in 2017, and *Colidextribacter massiliensis* is its only known species (Ricaboni et al., 2016). Wang et al. (2021) reported that the administration of olive fruit extracts improved antioxidant activity, which was associated with an increase in *Alloprevotella* sp. and a decrease in *Colidextribacter*. *Colidextribacter* was positively correlated with products of lipid peroxidation, such as malondialdehyde (MDA), and negatively correlated with antioxidant enzyme activities, such as total antioxidant activity (T-AOC), superoxide dismutase (SOD), and glutathione peroxidase (GSH-Px). Oxidative stress is a state of excessive reactive oxygen species, and an imbalance in antioxidant defense has been reported to be associated with obesity (Fernández-Sánchez et al., 2011). Decreased antioxidant defense markers, such as SOD and GSH-Px, and oxidative stress markers, such as MDA, have been observed in obese and diabetic individuals (Lee et al., 2015; Gunawardena et al., 2019). Yesilbursa et al. (2005) demonstrated that obesity is associated with enhanced lipid peroxidation and Orlistat, which is a drug designed to treat obesity by promoting weight reduction and lipid peroxidation. These observations suggest that one of the anti-obesity mechanisms of the gut microbiota may be mediated by antioxidant defense.

It is widely agreed that obesity and immunity are closely related. Specifically, enlargement of adipose tissue is associated with prominent inflammatory responses, such as infiltrating immune cells and increases in pro-inflammatory cytokines and adipokines (Sell et al., 2012). Oh et al. (2010) found that GPR120 functions as an omega-3 fatty acid receptor and mediates anti-inflammatory effects by repressing macrophage-induced tissue inflammation. Eicosapentaenoic acids, which are polyunsaturated free fatty acids such as omega-3, also inhibit high-fat/high-sucrose-induced inflammation in adipose tissue *via* GPR120 (Yamada et al., 2017). Indeed, GPR120 dysfunction leads to obesity in mice, and the genetic mutation of *GPR120*, which inhibits GPR120 signaling activity, has been observed in obese children and adults (Ichimura et al., 2012). Tanagho and Shohdy (2016) evaluated GPR120 as a potential target receptor for the treatment of obesity. In the present study, we found that LS and PT increased the level of *GPR120* compared to that in the HFD group. Moreover, improvements in adipose tissue inflammation were observed following administration of LS and PT in the HFD model. These results suggest that the effects of LS and PT administration on GPR120 and adipose tissue inflammation may be a possible mechanism of anti-obesity effect of LS and PT. We measured *GPR120* in colon tissue; however, it is necessary to conduct further studies to confirm the expression level of GPR120 in adipose tissue, and to clarify the association between GPR120 in adipose tissue and drugs mediated by the gut microbiota. In addition, further studies on morphological changes of mice colon or cecum will also help to understand the relationship between anti-obesity drugs and gut microbiota.

In this study, LS and PT administration did not affect GPR41 and GPR43. We only investigate the mRNA levels of *GPR41* and *GPR43* using RT-qPCR, but the Western blot is also widely used and may help to better understand the effect of LS and PT on GPR41 and GPR43. Further studies using various approaches including the Western blot are needed.

Next, we found that *Eubacterium_coprostanoligenes_group* was increased after LS and PT administration. Furthermore, ASV3566, corresponding to *Eubacterium_coprostanoligenes_group,* showed a significant correlation with GPR120, GPT, and TG. A recent study reported that *Eubacterium_coprostanoligenes_group* is one of the hub genera in the fecal micro-ecosystem of HFD-fed subjects and mediates the effect of HFD on dyslipidemia through sphingosine (Wei et al., 2021). The study also found an association of *Eubacterium_coprostanoligenes_group* with host lipid metabolism, such as GPT and TG. An HFD-fed mouse model showed a significant negative correlation of the *Eubacterium coprostanoligenes* group with changes in body weight, liver weight, and fasting glucose (Chen et al., 2021). *Eubacterium coprostanoligenes* was reported to reduce cholesterol to coprostanol (Freier et al., 1994). Oral administration of *E. coprostanoligenes* ATCC 51222 lowered the cholesterol concentration in germ-free mice and rabbits (Li et al., 1995, 1998). Overall, we presumed that enriching *Eubacterium_coprostanoligenes_group* and ASV3566 might contribute to the anti-obesity effect by participating in the regulation of GPR120 and metabolic disorders. Nevertheless, simple correlation result of this study may not be sufficient to establish the association between the anti-obesity effect of LS and PT on gut microbiota and GPR120. Further studies such as using *Gpr120*^−/−^ mice will be needed to elucidate sufficient associations among drugs, gut microbiota, and GPR120.

Treatment with LS for 1 year led to significant reductions in waist circumference and inflammatory markers such as high-sensitivity C-reactive protein and fibrinogen compared to placebo treatment (Redman and Ravussin, 2010). Treatment with PT for 13 weeks also led to significant reductions in waist circumference compared to placebo treatment (Kim et al., 2006). However, PT had an inflammatory effect. Moreover, the effects of LS and PT on gut microbiota have not been studied in humans. Further human studies identifying the effect of LS and PT on gut microbiota and their association with changes in fat deposition and inflammation would provide a more in-depth understanding of drug metabolism and involvement of gut microbiota in human metabolism. In addition, energy expenditure and activity levels were not measured in this study. These need to be measured in future studies. Research confirming the efficacy of various drugs using a mouse model can provide an important basis for applying drugs for therapeutic use in humans, and evidence for understanding drug metabolism.

In summary, we investigated the relationship between anti-obesity drugs and the gut microbiota. Our results demonstrated that anti-obesity drugs LS and PT induced changes in the gut microbiota composition in the HFD-induced obese mouse model. LS and PT have anti-obesity effects independent of the gut microbiota; however, some indicators such as visceral fat and GPT are affected by gut microbiota. In addition, LS and PT increase the level of GPR120 and alleviate inflammation in adipose tissue, which is influenced by the gut microbiota. Thus, we propose that anti-obesity drugs can affect the gut microbiota and that the synergistic actions of the gut microbiota can be expected to have an anti-obesity effect.

## Data availability statement

The datasets presented in this study can be found in online repositories. The names of the repository/repositories and accession number(s) can be found at: https://www.ddbj.nig.ac.jp/, DRA013284.

## Ethics statement

The animal study was reviewed and approved by the Institutional Animal Care and Use Committee (IACUC-2019-06187) of Dongguk University Ilsan Hospital.

## Author contributions

E-JS: formal analysis, visualization, and writing—original draft. NS: formal analysis and writing—original draft. SJ: resource and supervision. Y-DN and HK: conceptualization, supervision, and writing—review and editing. All authors contributed to the article and approved the submitted version.

## Funding

This work was supported by the Main Research Program of the Korea of Food Research Institute (KFRI) funded by the Ministry of Science and ICT (grant number E0170600-06), by National Research Fund of Korean Government (grant number 2019R1A6A3A01096837), and by a grant of the Korea Health Technology R&D Project through the Korea Health Industry Development Institute (KHIDI), funded by the Ministry of Health and Welfare, Republic of Korea (grant number HF20C0020).

## Conflict of interest

The authors declare that the research was conducted in the absence of any commercial or financial relationships that could be construed as a potential conflict of interest.

## Publisher’s note

All claims expressed in this article are solely those of the authors and do not necessarily represent those of their affiliated organizations, or those of the publisher, the editors and the reviewers. Any product that may be evaluated in this article, or claim that may be made by its manufacturer, is not guaranteed or endorsed by the publisher.

## Supplementary material

The Supplementary material for this article can be found online at: https://www.frontiersin.org/articles/10.3389/fmicb.2022.1109651/full#supplementary-material

## Footnotes

^1^ http://huttenhower.sph.harvard.edu/galaxy
