# Supplementary material for: Lorcaserin and phentermine exert anti-obesity effects with modulation of the gut microbiota
Source: Front Microbiol. 2023 Jan 5;13:1109651. doi: 10.3389/fmicb.2022.1109651 (PMC9849812; doi:10.3389/fmicb.2022.1109651)
Supplement: Supplementary file 1 [file Data_Sheet_1.docx]

Supplementary Material

# Supplementary Tables and Figures

## Supplementary Tables

| **Target** | **Primer Sequence (5’ to 3’)** |
| --- | --- |
| GAPDH | F: TGA TGA CAT CAA GAA GGT GGT GAA  R: TCC TTG GAG GCC ATG TAG GCC AT |
| GPR41 | F: GGG GTC GAT ACA AGA GT  R: CTG GCG GAG CTA CGT GCT |
| GPR43 | F: ACA GTG GAG GGG ACC AAG AT  R: GGG GAC TCT CTA CTC GGT GA |
| GPR120 | F: GTG CCG GGA CTG GTC ATT GTG  R: TTG TTG GGA CAC TCG GAT CTG G |
| IL-10 | F: CAG GAC TTT AAG GGT TAC TTG  R: ATT TTC ACA GGG GAG AAA TC |
| IL-13 | F: TGA GGA GCT GAG CAA CAT CAC ACA  R: TGC GGT TAC AGA GGC CAT GCA ATA |
| TNF-α | F: GAC CCT CAC ACT CAG ATC ATC TTC T  R: CCA CTT GGT GGT TTG CTA CGA |
| IL-1β | F: TGC CAC CTT TTG ACA GTG ATG  R: AAG GTC CAC GGG AAA GAC AC |
| IL-6 | F: CAT CCT CGA CGG CAT CTC AG  R: GCT CTG TTG CCT GGT CCT |
| MCP-1 | F: TTA AAA ACC TGG ATC GGA ACC AA  R: GCA TTA GCT TCA GAT TTA CGG GT |

## Supplementary Figures

#
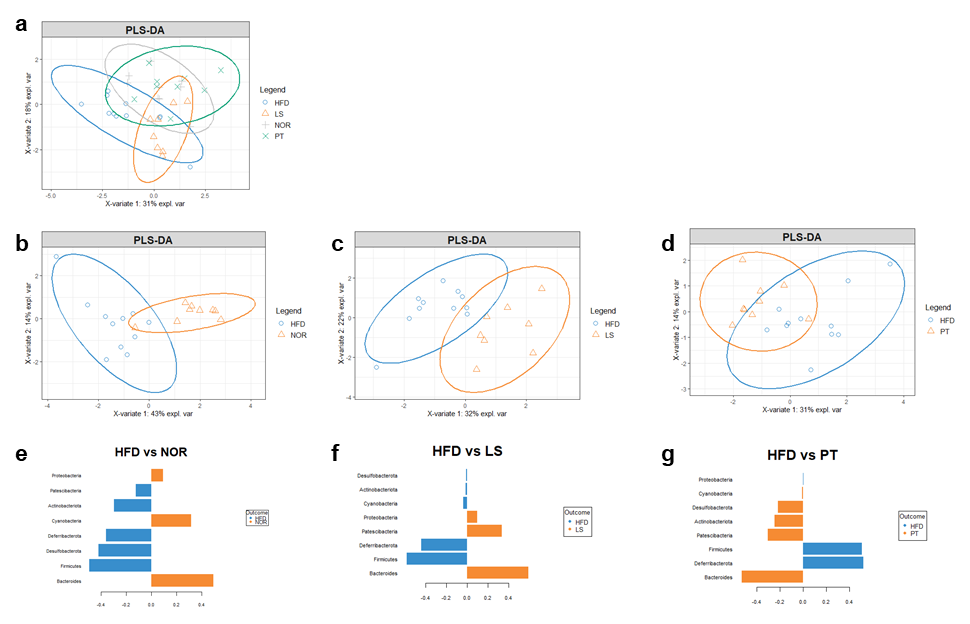


# Supplementary Figure 1, At phylum level, PLS-DA (a) score plot of all groups, (b) score plot of NOR and HFD groups, (c) score plot of HFD and LS groups, (d) score plot of HFD and PT groups. PLS-DA (e) loading plot of NOR and HFD groups, (f) loading plot of HFD and LS groups, and (g) loading plot of HFD and PT groups. NOR, normal diet; HFD, high-fat diet; LS, lorcaserin; PT, phentermine.

#
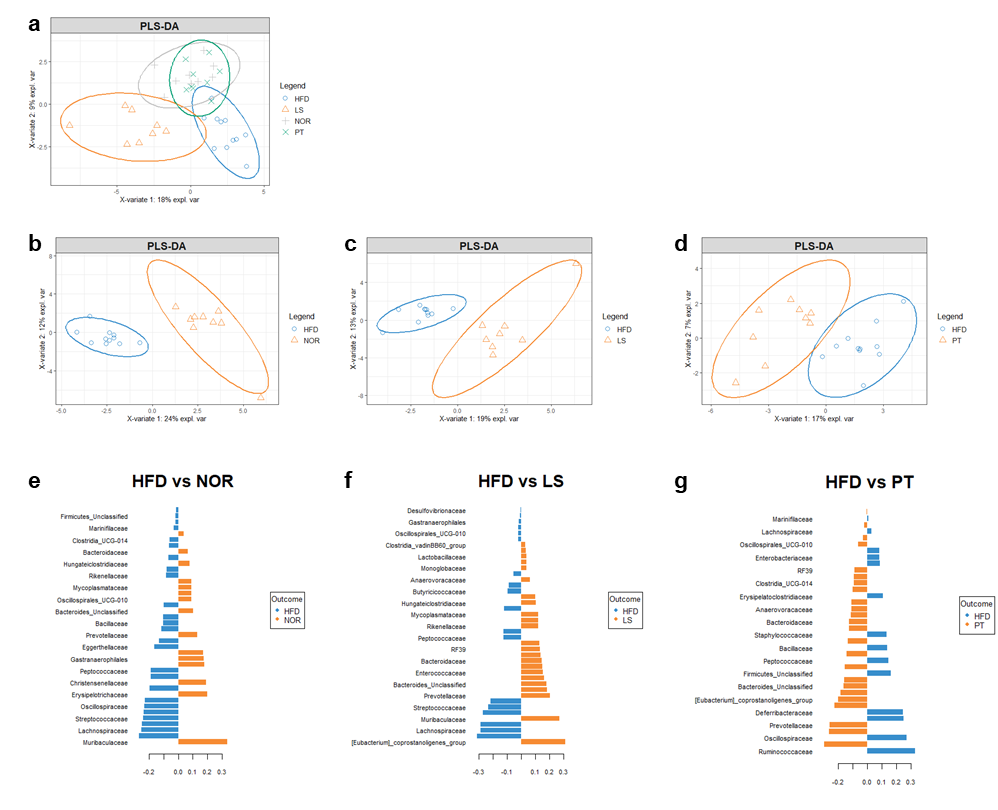


# Supplementary Figure 2, At family level, PLS-DA (a) score plot of all groups, (b) score plot of NOR and HFD groups, (c) score plot of HFD and LS groups, (d) score plot of HFD and PT groups. PLS-DA (e) loading plot of NOR and HFD groups, (f) loading plot of HFD and LS groups, and (g) loading plot of HFD and PT groups. NOR, normal diet; HFD, high-fat diet; LS, lorcaserin; PT, phentermine.

#
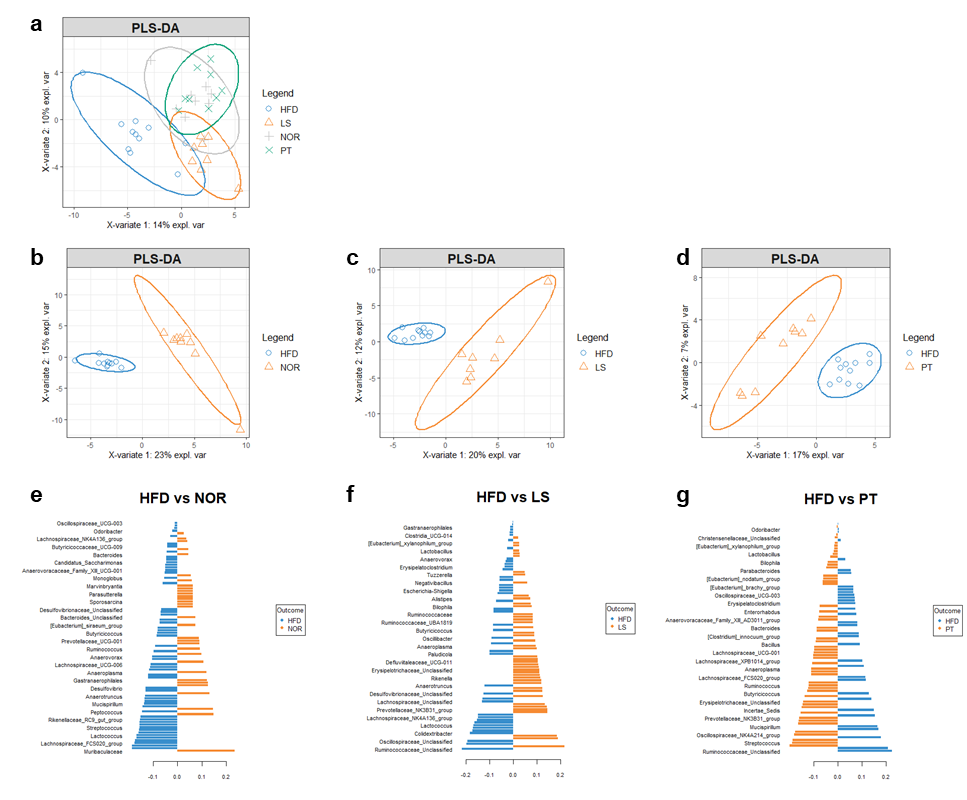


# Supplementary Figure 2, At genus level, PLS-DA (a) score plot of all groups, (b) score plot of NOR and HFD groups, (c) score plot of HFD and LS groups, (d) score plot of HFD and PT groups. PLS-DA (e) loading plot of NOR and HFD groups, (f) loading plot of HFD and LS groups, and (g) loading plot of HFD and PT groups. NOR, normal diet; HFD, high-fat diet; LS, lorcaserin; PT, phentermine.


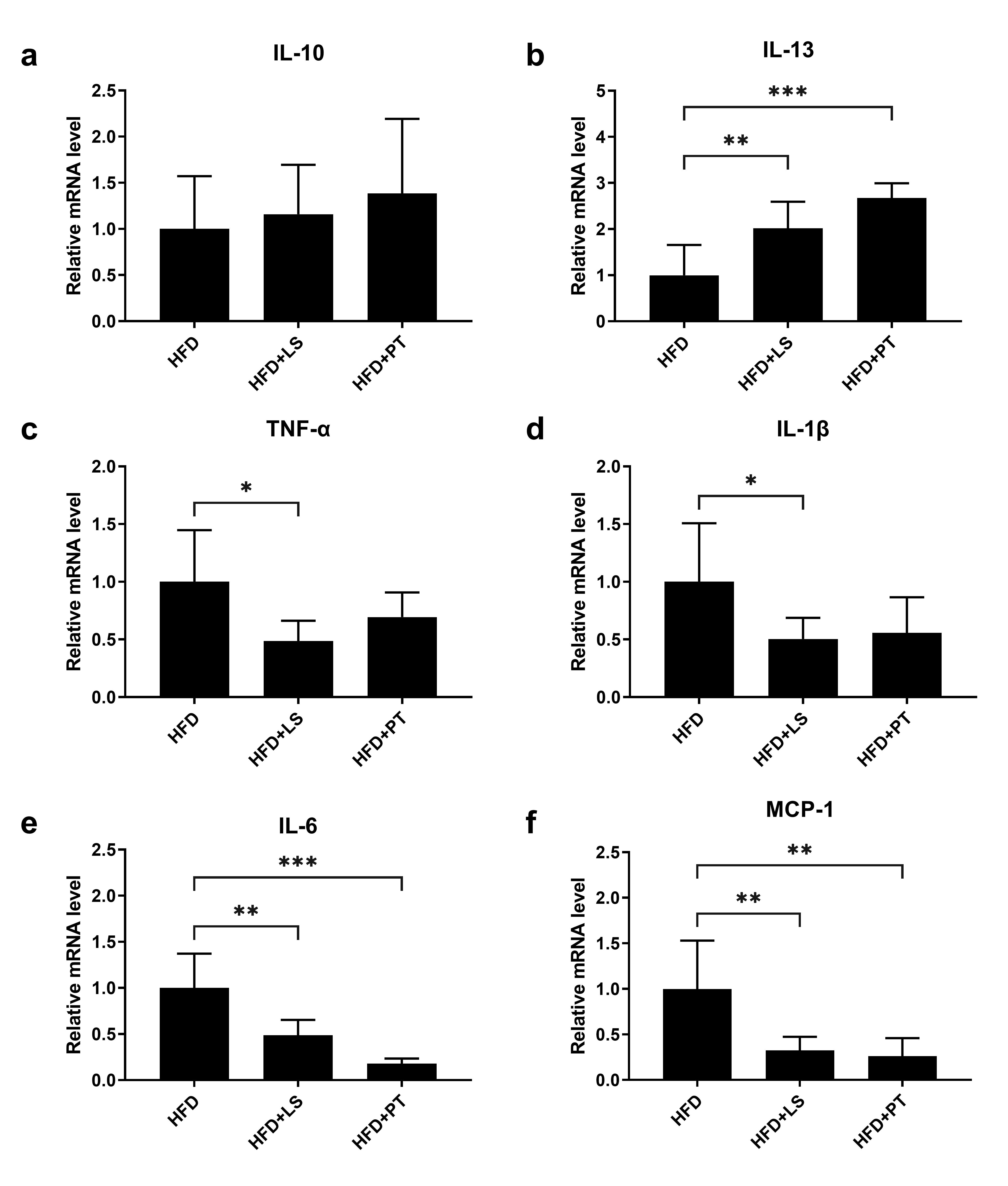


**Supplementary Figure 4 Levels of inflammatory cytokines in adipose tissue.** Expression of anti-inflammatory cytokines (a) IL-10 and (b) IL-13, and pro-inflammatory cytokines (c) TNF-α, (d) IL-1β, (e) IL-6, and (f) MCP-1 mRNA detected using real-time PCR. Statistical significance assessed by one-way ANOVA, **P* < 0.05, compared to the HFD group. HFD, high-fat diet; LS, lorcaserin; PT, phentermine.
